# Glymphatic system and psychiatric disorders: need for a new paradigm?

**DOI:** 10.3389/fpsyt.2025.1642605

**Published:** 2025-12-04

**Authors:** Tommaso Barlattani, Alessandro Cavatassi, Antony Bologna, Valentina Socci, Edoardo Trebbi, Maurizio Malavolta, Alessandro Rossi, Vassilis Martiadis, Carmine Tomasetti, Domenico De Berardis, Giorgio Di Lorenzo, Francesca Pacitti

**Affiliations:** 1 Department of Biotechnological and Applied Clinical Sciences (DISCAB), University of L’Aquila, L’Aquila, Italy; 2 Department of Public Health and Infectious Diseases, “La Sapienza” University of Rome, Rome, Italy; 3 Department of Mental Health, Asl Napoli 1 Centro, Naples, Italy; 4 National Health Service, Department of Mental Health, Psychiatric Service of Diagnosis and Treatment, Hospital G. Mazzini, Teramo, Italy; 5 Department of Systems Medicine, University of Rome Tor Vergata, Rome, Italy; 6 Istituto di Ricovero e Cura a Carattere Scientifico (IRCCS) Fondazione Santa Lucia, Rome, Italy

**Keywords:** glymphatic system, mental disorders, diffusion tensor imaging, aquaporin-4, astrocytes, cerebrospinal fluid, extracellular fluid, sleep

## Abstract

Psychiatric disorders like depression, bipolar disorder, schizophrenia, and post-traumatic stress Disorder have conventionally theorized on alterations in neurotransmitters, receptor pharmacodynamics, and neural connectivity. However, recent research points to a complementary framework involving the glymphatic system, a specialized glial lymphatic pathway that removes metabolic waste products, particularly during deep sleep, through the coordinated action of cerebrospinal fluid, interstitial fluid, and the aquaporin 4 channels of astrocytes. When the glymphatic network is compromised, neurotoxic proteins, such as beta-amyloid and tau, and inflammatory mediators can accumulate, potentially exacerbating insomnia, inflammation, and circadian disturbances. These same processes often occur in psychiatric disorders, fueling oxidative stress, neuroinflammation, and cognitive decline. New neuroimaging methods, such as diffusion tensor imaging and the analysis Along the Perivascular Space, ALPS, index, allow clinicians and researchers to quantify perivascular flow deficits *in vivo*. Preliminary evidence suggests that enhancing glymphatic function by improving sleep architecture, supporting astrocyte health, or scheduling drug delivery based on circadian fluctuations may offer clinical benefits. Here, we present an overview of glymphatic biology, examine its relevance to psychiatric pathophysiology, highlight findings from emerging neuroimaging studies, and consider ways modulating glymphatic flow may improve psychiatric pharmacotherapy.

## 1 Introduction

### 1.1 Background and rationale

**Figure 1:**
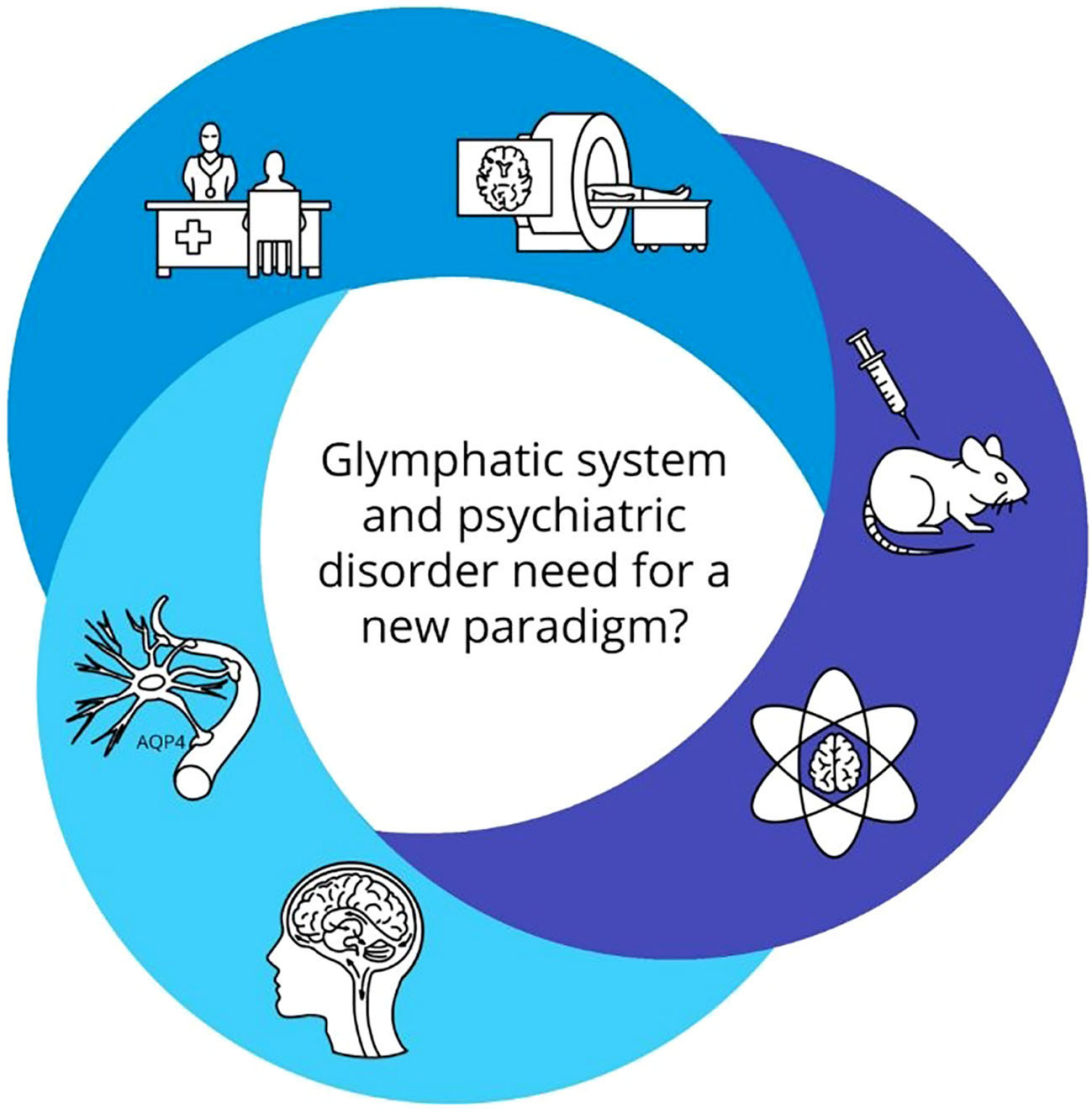
A new integrated and pragmatic theoretical model is essential for clinical application, diagnosis, prevention, and treatment, by correlating noninvasive neuroimaging data with alterations in the neuro-glio-vascular microenvironment and the broader macroenvironment. This framework helps explain the clinical symptoms observed in patients, murine models, and other experimental models with degenerative glymphatic changes.

Traditional neuron−centric models, focused on neurotransmitters, receptor signaling, and connectivity, do not fully capture the complexity of major psychiatric disorders (1–3). Currently, the attention has shifted to processes that are geared by brain systems that handle sleep patterns, inflammation, and metabolism in understanding and improving these complex conditions of psychiatric disorders (4–8). One of the systems that handles these processes of maintaining brain homeostasis is the glymphatic system. The key mechanism of this system is that it uses the organized network of perivascular pathways extending from the vasculature to push and circulate the cerebrospinal fluid (CSF) from the subarachnoid spaces to the brain parenchyma via specialized structures called aquaporin-4 (AQP4) water channels at the end feet of the astrocytic cells (9–11) **Figure 1**.

#### 1.1.1 CSF production and entry into brain parenchyma

Cerebrospinal fluid (CSF) is produced predominantly by the choroid plexus and flows from the ventricular system to the subarachnoid space (12, 13). From there, CSF enters the brain parenchyma mainly along peri−arterial, Virchow–Robin, spaces that accompany penetrating arteries, where it exchanges with interstitial fluid (ISF) through aquaporin−4 (AQP4)−rich astrocytic endfeet (14–16). In addition, local transependymal/subependymal exchange can contribute under specific physiological or pathological conditions (17). Outflow proceeds toward perivenous spaces, dural venous sinuses, and meningeal lymphatic vessels, ultimately draining to head and cervical, especially deep cervical, lymph nodes (18–22).

#### 1.1.2 Drivers of perivascular exchange

Perivascular CSF–ISF exchange is driven by arterial pulsatility, slow vasomotion generated by vascular smooth muscle cells, like the intramural periarterial drainage (IPAD) mechanism, and respiratory oscillations (14, 23, 24). Within the neuro−glio−vascular unit, AQP4 polarization at astrocytic endfeet lowers hydraulic resistance and supports efficient clearance (15, 25).

#### 1.1.3 Sleep in contex

Deep non−REM sleep transiently expands the extracellular space and enhances convective flow, wherea fragmented or insufficient sleep can diminish clearance capacity (16, 26–28). However, sleep outstand as one of several modulators alongside vascular, respiratory, and astroglial factors, rather than the sole driver of glymphatic clearance.

#### 1.1.4 Emerging *in vivo* biomarkers

Diffusion MRI methods, particularly Diffusion Tensor Imaging, Analysis Along the Perivascular Space (DTI−ALPS), together with BOLD−CSF and arterial spin labeling (ASL), provide noninvasive readouts of perivascular transport and related hemodynamics (17, 29, 30). Early studies in psychiatric cohorts associate altered glymphatic metrics with cognitive an affective symptom burden (31–35).

#### 1.1.5 Scope and article structure

We therefore evaluate glymphatic biology with emphasis on CSF production/entry routes and the main flow drivers, synthesize neuroimaging evidence across psychiatric conditions, and outline a glymphatic−oriented therapeutic perspective integrating sleep architecture, astrocyte/AQP4 health, vascular control, and dosing−time alignment. After this Introduction, we first present a biological overview (Section 1.2) and clinical relevance (Section 1.3); we then describe the Methods, followed by neuroimaging characteristics, pharmacotherapy implications, and a concise one−paragraph conclusion.

### 1.2 Overview of the glymphatic system

#### 1.2.1 CSF production, routes of entry, and outflow

The glymphatic pathway links CSF dynamics to ISF exchange: CSF is produced predominantly by the choroid plexus, travels through the ventricles to the subarachnoid space, and then enters the parenchyma primarily along Virchow–Robin spaces where it mixes with ISF via AQP4−enriched astrocytic endfeet (12–16, 36). In addition to this perivascular entry, transependymal/subependymal exchange can contribute under specific physiological or pathological conditions (17). Clearance proceeds along perivenous routes toward dural venous sinuses and meningeal lymphatic vessels, with downstream drainage to head and cervical, especially deep cervical, lymph nodes (18–20, 22, 37).

#### 1.2.2 Forces that drive glymphatic transport

Convective CSF–ISF exchange is propelled by a combination of arterial pulsatility, slow vasomotion generated by vascular smooth muscle cells, the IPAD mechanism, and respiratory oscillations (14, 23, 24, 38). Noradrenergic tone modulates the microarchitecture of non−REM sleep and the underlying vasomotion that supports bulk flow, providing a physiological link between arousal state and clearance efficiency (39). Within this framework, meningeal and dural lymphatic conduits complete the loop by returning solutes to extracranial lymph nodes (18, 20, 22, 40).

#### 1.2.3 Astrocytes, AQP4 polarization, and neuroimmune crosstalk

Astrocytic endfeet organize low−resistance perivascular conduits through AQP4 polarization (41); stress, inflammation, and vascular insults can mislocalize AQP4, heighten astrocyte reactivity, and amplify microglial signaling, thereby reducing perivascular exchange and perturbing glutamate and metabolic homeostasis (10, 15, 25, 42–45). These glial changes, by raising hydraulic resistance and altering neurovascular coupling, are expected to dampen glymphatic throughput and set the stage for neuroinflammatory feedback (1, 24, 46, 47).

##### 1.2.3.1 Sleep as one modulator among several

Deep non−REM sleep transiently expands the extracellular space and enhances convective flow, whereas sleep fragmentation or insufficiency can blunt this effect (16, 26). Despite its importance, sleep emerges as one of many factors influencing glymphatic function, along with IPAD vasomotricity, arterial pulsatility, respiration, and astroglial polarity.

##### 1.2.3.2 Posture, respiration, and practical considerations

Experimental work in rodents indicates that lateral recumbency favors tracer clearance compared with supine or prone postures, likely by reducing venous outflow resistance; most reports specify “lateral” without a consistent right/left preference, and the principal comparison is lateral versus non−lateral positions (14, 16). Respiratory oscillations additionally entrain CSF movement and interact with cardiac pulsatility to shape perivascular transport (24).

##### 1.2.3.3 Summary and link to psychiatric relevance

In sum, the glymphatic system is a paravascular–lymphatic interface governed by CSF production and routing, vascular and respiratory forcing, and astrocyte/AQP4 biology, with efflux through meningeal lymphatics to deep cervical nodes (13, 14, 18, 20, 22). Because sleep disruption, inflammation, and vascular stiffening converge on these same mechanisms, they represent plausible levers through which psychiatric conditions may alter clearance and, reciprocally, be exacerbated by impaired removal of neurotoxic and inflammatory mediators (15, 24).

### 1.3 Relevance for psychiatric disorders

Glymphatic dysfunction is clinically relevant to psychiatry because impaired CSF–ISF exchange mislocalization of AQP4, and altered vascular/respiratory forcing can promote retention of neurotoxic proteins and inflammatory mediators, disrupt neuro−glio−vascular coupling, and degrade synaptic and cognitive integrity, mechanisms that map onto symptom clusters observed across mood, psychotic, stress−related, substance−use, and neurodevelopmental disorders (4, 9, 14–16, 24, 48–50).

#### 1.3.1 Pathways linking glymphatic impairment to psychiatric phenotypes

Direct, clearance−centric, pathway. When perivascular flow slows, removal of β−amyloid, tau, reactive oxygen species, and cytokines is diminished, fostering neuroinflammation and synaptopathy that manifest as anergia, anhedonia, impaired executive control, and negative symptoms (14, 16, 24, 48, 51). In parallel, elevated interstitial metabolites perturb astrocytic glutamate handling and neurovascular coupling, compounding cognitive−affective dysfunction (15, 52).

Sleep–circadian−mediated pathway. Insomnia, hyperarousal, and circadian misalignment curtail deep non−REM−linked expansion of the extracellular space and lower convective flux; over time, this amplifies inflammatory signaling and symptom persistence (5, 6, 16, 26). Noradrenergic dynamics during sleep modulate slow vasomotion, a driver of glymphatic flow, thus arousal level and clearance efficiency are directly connected (39, 53).

Vascular–metabolic–immune pathway. Arterial stiffening, metabolic dysregulation, and systemic inflammation degrade pulsatility and IPAD vasomotion, mislocalize AQP4, and raise hydraulic resistance, thereby compounding clearance failure (4, 15, 23, 25, 40, 54). These changes also interact with endothelial dysfunction and microglial activation to sustain low−grade neuroinflammation relevant to chronic mood and psychotic illness (24, 55–57).

#### 1.3.2 Neuroimaging evidence and biomarkers of relevance

Diffusion MRI approaches quantify paravascular transport *in vivo* (17, 58). The DTI−ALPS index measures water diffusion along perivenular spaces and serves as a proxy of glymphatic transport efficiency; moreover, related signals from BOLD−CSF and ASL could provide complementary information on CSF pulsatility and cerebral perfusion (17, 24, 29, 30, 59). Across psychiatric cohorts, lower ALPS values and abnormal BOLD−CSF coupling associate with cognitive deficits, fatigue, and disease duration, supporting a clearance component beyond neurotransmitter models (31–34). Importantly, ALPS reductions have been observed even with minimal antipsychotic exposure, suggesting that glymphatic alterations are not solely medication effects (60, 61).

#### 1.3.3 Structural and inflammatory correlates strengthen biological plausibility

Choroid plexus enlargement and systemic inflammatory/oxidative markers have been linked to reduced glymphatic indices in depression, indicating a nexus between CSF production interfaces, immune trafficking, and paravascular clearance (62). Moreover, coupling ALPS with regional cerebral blood flow improves classification in stimulant−use cohorts, consistent with intertwined vascular and perivascular mechanisms (63).

#### 1.3.4 Disorder−specific summaries

Major depressive disorder (MDD). MDD frequently features sleep fragmentation, elevated inflammatory burden, and cognitive inefficiency. Imaging studies report lower ALPS indices correlating with fatigue and cognitive symptoms, alongside inflammatory signatures and choroid plexus changes that implicate impaired perivascular transport (32, 34, 62, 64). Drug−naïve somatic depression may show increased ALPS, possibly compensatory, highlighting state/stage heterogeneity (65).

Bipolar disorder (BD). In BD, erratic sleep–wake cycles, circadian dysregulation, and metabolic stress converge on glymphatic inefficiency. Diffusion metrics, including free−water alterations, suggest extracellular fluid shifts and astroglial involvement beyond purely neurotransmitter−based accounts (35). Frontal pole atrophy has been associated with lower ALPS, reinforcing structural–functional coupling (66). Clinically, stabilizing sleep/circadian timing and reducing inflammatory load are predicted to mitigate neuroprogression and cognitive decline (6, 67, 68).

Schizophrenia/psychosis. Characteristic abnormalities in sleep architecture, like reduced slow−wave activity and frequent awakenings, coincide with ALPS alterations and reduced BOLD−CSF clearance in early psychosis, supporting an intrinsic clearance component linked to cognitive/negative symptoms (31, 33, 69). Observations in individuals minimally exposed to antipsychotics indicate that these glymphatic changes are present early and are unlikely to be medication artifacts (60, 61). Preliminary ALPS reductions have also been noted in acutely hospitalized young adults during a first psychotic episode (70).

Post−traumatic stress disorder (PTSD). Persistent hyperarousal and elevated nocturnal noradrenergic tone can suppress slow vasomotion and glymphatic throughput. ALPS index abnormalities could parallel symptom severity, suggesting a pathophysiological loop in which impaired clearance maintains stress−related molecular signatures, augments fear memory, and perpetuates sleep fragmentation (16, 34, 39).

Substance use disorders (SUD). Alcohol and stimulants disrupt sleep architecture and perivascular flow, entraining inflammatory loops that worsen cognition and relapse risk (71, 72). Diffusion and cerebral blood flow coupling analyses reveal multi−system involvement, and glymphatic metrics correlate with addiction trajectories and classification performance in methamphetamine cohorts (63, 73, 74).

Neurodevelopmental disorders (ADHD/ASD). Atypical sleep and ALPS−indexed dysfunction in youth suggest early contributions of impaired CSF–ISF exchange to attentional, executive, and social−communication trajectories (75, 76). Adult ADHD also shows reduced ALPS tracking cognitive performance (77). In pediatric ASD, a positive association between ALPS and age suggest a delayed or altered maturation of perivascular exchange in ASD (75). These associations accord with broader evidence of astrocyte−mediated circuit refinement and the vulnerability of AQP4 polarity to stress/inflammation during sensitive developmental windows (78–81).

#### 1.3.5 Aging and risk modifiers

Vascular aging, APOE−related glial vulnerability, and chronic sleep loss synergistically lower glymphatic output and raise neurodegenerative risk, offering a parsimonious link between late−life psychiatric burden, cognitive decline, and clearance failure (14, 82–85).

#### 1.3.6 Clinical implications and empirically testable directions

Cross−sectional: severity of insomnia, inflammatory load, and arterial stiffness will independently predict lower DTI−ALPS values and weaker BOLD−CSF coupling after adjustment for age, sex, head motion, and medication exposure. Longitudinal: within−person increases in slow−wave activity or reductions in systemic inflammatory markers (e.g., C−reactive protein) will precede, and possibly correlate with, subsequent alterations in ALPS/BOLD−CSF signals.

Interventional: CBT−I or circadian stabilization, like structured light–dark exposure, aerobic exercise, and anti−inflammatory/astroglial−supportive strategies will increase ALPS/BOLD−CSF readouts in parallel with symptomatic improvement. Conversely, interventions that enhance slow−wave sleep, restore AQP4 polarity, and improve vascular drivers (pulsatility/IPAD) should improve glymphatic metrics and clinical outcomes (17, 29, 30, 86).

## 2 Materials and methods

### 2.1 Review design

This work is a narrative review that adopts selected items from the PRISMA 2020 recommendations, information sources, search strategy, eligibility criteria, study selection, and data items, but does not constitute a full systematic review. No protocol was registered; no formal risk−of−bias tool was applied; and no meta−analysis was conducted owing to heterogeneity in study designs, imaging metrics, and populations (87).

### 2.2 Information sources and search strategy

We searched ERIC, MEDLINE, PsycARTICLES, PsycINFO, Scopus, and PubMed without date limits; English−language records only were considered. The final comprehensive database search was completed on May 31, 2025, and an update search was performed through August 2025 to capture late−breaking publications now reflected in **Table 1**. To contextualize emerging concepts, we also screened preprint servers (e.g., SSRN) and flagged their status explicitly in tables. To ensure comprehensive coverage of the topic, the research strategy included a broad set of English-language keywords identified and combined using Boolean operators (AND, OR, NOT). The search terms included “glymphatic system,” “glymphatic,” “brain lymphatic,” “cerebrospinal fluid,” “CSF,” “brain clearance,” “interstitial fluid,” “astroglial,” “neurovascular,” “DTI,” “ALPS Index,” “BOLD CSF,” “arterial spin labeling/ASL,” “free water,” “MRS/macromolecule” “psychiatric disorders,” “mental disorders,” “psychiatric conditions,” “mood disorders,” “affective disorders,” “depressive disorders,” “depression,” “bipolar disorder,” “anxiety disorder,” “panic disorder,” “phobia,” “obsessive compulsive disorder,” “OCD,” “attention deficit hyperactivity disorder,” “ADHD,” “autism spectrum disorder,” “ASD,” “schizophrenia,” “psychotic disorders,” “sleep disorders,” “sleep-wake disorders,” “insomnia,” “personality disorders,” “borderline personality disorder,” “narcissistic personality disorder,” “antisocial personality disorder,” “substance use disorder,” “SUD,” “trauma,” “PTSD,” “post-traumatic stress disorder,” “eating disorders,” “feeding disorders,” “bulimia,” “anorexia,” “binge eating,” “stress,” “distress,” “adjustment disorder,” and “mental illness.”

**Table 1:** Summary of human neuroimaging studies examining glymphatic-related MRI measures across psychiatric and neurodevelopmental disorders, including key aims, methods, findings, and clinical correlates.

Author(s)	Year	Study	Location	Sample	Diagnosis	Key aims	Main measures	Key findings	Clinical/biological correlates
Li et al.	2022	Children with autism spectrum disorder present with glymphatic system dysfunction highlighted by DTI−ALPS	China	30 ASD, 25 HC	ASD	Test ALPS differences and age effects	DTI−ALPS	Lower ALPS in ASD; ALPS positively correlated with age in ASD	Developmental trajectory implications
Chen et al.	2023	Evaluation of glymphatic function in children with ADHD	China	47 ADHD (drug−naïve), 52 HC	ADHD (child)	Evaluate GS impairment in ADHD	DTI−ALPS; Conners	Lower ALPS in ADHD *vs* HC	Links to attention/executive symptoms
Fang et al.	2025	Glymphatic dysfunction in adult ADHD: relationship to cognitive performance	USA	41 ADHD−adult, 123 HC	ADHD (adult)	Test ALPS–cognition associations	DTI−ALPS; CVLT; symptom scales	Lower ALPS relates to memory/executive deficits	Cognitive impairment linkage
Ueda et al.	2024	Glymphatic system dysfunction in mood disorders	Japan	58 BD, 66 HC	Bipolar disorder	Test glymphatic alterations in BD	DTI−ALPS; FWI; HAMD; YMRS	No robust ALPS difference; increased free water (callosal) suggests neuroinflammation	Fluid/inflammatory signatures
Kikuta et al.	2025	Association between frontal−pole atrophy and glymphatic dysfunction in BD	Japan	MRI cohort (BD)	Bipolar disorder	Link regional atrophy to glymphatic metrics	DTI−ALPS; cortical thickness	Frontal−pole atrophy associates with lower ALPS	Neurodegeneration−clearance nexus.
Yang et al.	2024	Glymphatic function and white−matter alterations in MDD (reviewed/quantified)	China	35 MDD, 23 HC	MDD	Assess ALPS in MDD & white−matter microstructure	DTI−ALPS; DTI metrics; HAMD/HAMA/MoCA	Lower ALPS in MDD; associations with WM abnormalities	Cognitive & anxiety burden; WM changes.
Bao et al.	2025	Glymphatic dysfunction in MDD revealed by DTI−ALPS: correlation with fatigue	China	46 MDD, 55 HC	MDD	Test ALPS *vs* fatigue/depression	DTI−ALPS; HAMD; Chalder Fatigue	Lower ALPS in MDD; links to fatigue severity	Fatigue pathophysiology
Gong et al.	2025	Glymphatic function & ChP volume linked to systemic inflammation/oxidative stress in MDD	China	665 MDD, 338 HC	MDD	Relate ALPS & ChP to immune markers	DTI−ALPS; automated ChP; blood indices	Larger ChP & lower ALPS in MDD; ALPS correlates with NLR/PLR/SII in MDD compared to HC	Immune–CSF–clearance axis.
Chen et al.	2025	Glymphatic dysfunction associated with cortisol dysregulation in MDD	China	MDD cohort + HC	MDD	Test ALPS *vs* HPA/cortisol	DTI−ALPS; diurnal cortisol	Lower ALPS correlates with cortisol dysregulation	HPA–glymphatic coupling
Liang et al.	2025	Inflammation and psychomotor retardation in depression: moderating role of GS	China	67 MDD, 67 HC	MDD	Does ALPS moderate inflammation–PMR	DTI−ALPS; hsCRP; PMR; motor FC	Lower ALPS magnifies hsCRP–PMR link; ALPS moderates motor−network effects	Inflammation–motor circuit coupling.
Deng et al.	2025	Increased GS activity & thalamic vulnerability in drug−naïve somatic depression	China	272 total (SMD, PMD, HC)	MDD (SMD/PMD)	Compare ALPS among subgroups	DTI−ALPS; VBM thalamus	Higher ALPS (awake) in MDD—esp. SMD; ALPS–thalamus volume positive correlation	State−dependent activity; thalamic link.
Tao et al.	2025	Altered DMN and glymphatic function in insomnia with depression	China	60 CID+MDD, 52 CID−only, 56 HC	CID ± MDD	Examine DMN–glymph coupling	DTI−ALPS; rs−fMRI; PSQI/HAMD	DMN disruption parallels ALPS changes in CID comorbid with MDD	Sleep–glymph–mood interactions
Korann et al.	2025	Dysregulation of the glymphatic system in psychosis with minimal antipsychotics	Canada	13 psychosis (minimally exposed), 123 HC	Psychotic spectrum	Test ALPS in early/limited exposure	DTI−ALPS; EPS scales	Lower ALPS *vs* HC despite minimal exposure	Intrinsic alteration beyond medication.
Tu et al.	2024	Glymphatic dysfunction in schizophrenia associates with cognitive impairment	China	43 SZ, 108 HC	Schizophrenia	Link ALPS to cognition/symptoms	DTI−ALPS; SAPS/SANS; cognition	Lower ALPS in SZ; associations with cognition	Negative/cognitive symptom load.
Abdolizadeh et al.	2024	Glymphatic evaluation with macromolecules & DTI−ALPS in SZ	Canada	103 SZ, 47 HC	Schizophrenia	Test macromolecule diffusivity *vs* ALPS	DTI−ALPS; 1H−MRS macromolecules	Lower ALPS in SZ; macromolecules not different	Candidate clearance deficit.
Hua et al.	2025	Reduced glymphatic clearance in early psychosis	China	Early psychosis cohort	Early psychosis	Assess early−stage clearance	BOLD–CSF coupling; ALPS	Reduced clearance early; links to symptoms	Early biomarker potential
Wu et al.	2025	GS dysfunction correlates with gut dysbiosis & cognition in SZ	China	SZ + HC; microbiome	Schizophrenia	Integrate microbiome and ALPS	DTI−ALPS; 16S microbiome; cognition	ALPS reduction tracks dysbiosis and cognitive loss	Microbiome–glymph axis.
Shao et al.	2024	Linking ALPS with cortical microstructure in PTSD	China	67 male veterans	PTSD	Detect early neurodegenerative signal	DTI−ALPS; cortical MD; neurocognitive scales	Lower ALPS associates with increased cortical MD	Neurodegeneration−vulnerability
Dai et al.	2024	DTI−ALPS and cognition in alcohol use disorder	China	40 AUD, 40 HC	Alcohol use disorder	Test ALPS–cognition	DTI−ALPS; MoCA/MMSE	Lower ALPS correlates with cognitive deficits	Memory/executive burden.
Wang et al.	2023	Glymphatic function in heroin dependence on methadone	China	51 MMT, 48 HC, 20 HD	Opioid use	Relate GS to addiction/relapse	DTI−ALPS; clinical outcomes	ALPS associates with outcomes during MMT	Relapse risk & inflammation.
Cheng et al.	2025	ALPS–CBF coupling in methamphetamine dependence	China	46 METH, 46 HC	Methamphetamine	Improve classification	DTI−ALPS; ASL−CBF; ML models	ALPS–CBF coupling improves discrimination	Vascular–perivascular interplay
Barlattani et al.	2025	GS dysfunction in young adults hospitalized for acute psychosis (pilot)	Italy	First−episode cohort	Acute psychosis	Feasibility and signal	DTI−ALPS; SCADIS; PSQI, MoCA	Preliminary ALPS abnormality during acute psychosis	Acute−state biomarker.
Ma et al. (preprint)	2025	Glymphatic–rumination relationship in MDD	China/HK	51 MDD, 45 HC	MDD	Test ALPS rumination/depression	DTI−ALPS; RRS/HAMD; static/dynamic FC; PET maps	Lower ALPS associates with rumination; FC/dFC and neurotransmitter maps link ALPS, rumination and depression	Candidate cognitive mediator

ALPS, Analysis Along the Perivascular Space; CP(V), choroid plexus (volume); DMN, default mode network; d/sFC, dynamic/static functional connectivity; PMR, psychomotor retardation; CID, chronic insomnia disorder.

### 2.3 Eligibility criteria

We included experimental, observational, and theoretical articles that examined or discussed glymphatic−related physiology CSF/ISF exchange, perivascular flow, ALPS/BOLD−CSF/ASL markers, AQP4/astroglia, meningeal lymphatics in relation to psychiatric conditions mood, psychotic, anxiety/trauma−related, substance−use, neurodevelopmental. Human and relevant preclinical studies were eligible. Studies were also included when glymphatic metrics were explicitly linked to: endocrine measures, microbiome features, structural interfaces, or transdiagnostic constructs. Preprints were cite for background and clearly identified; they were not pooled with peer−reviewed MRI outcomes in any quantitative synthesis.

### 2.4 Study selection

Two reviewers independently screened titles/abstracts and then full texts against prespecified criteria; disagreements were resolved by consensus with a third reviewer. Duplicates across databases were removed before screening using a reference−management workflow. The August 2025 update search was screened with the same procedure and labeled by publication status (peer−reviewed *vs*. preprint).

### 2.5 Data extraction and synthesis approach

For each record, two reviewers extracted study design; sample and diagnosis; imaging method (DTI−ALPS, BOLD−CSF, ASL, free water, MRS/other) and primary glymphatic metrics; direction of effect versus controls; clinical correlates (symptoms, cognition, disease duration, inflammatory/oxidative markers); medication exposure; and potential confounders (sleep measures, time−of−day/circadian phase, vascular/metabolic comorbidity). Additional fields captured update−driven correlates (HPA/cortisol assay and timing; gut microbiome pipeline and diversity/taxa summaries; choroid plexus/frontal pole volumes; rumination scales). Publication status was recorded. Extraction used a piloted template. Given heterogeneity, we conducted a structured narrative synthesis, grouping findings by mechanistic pathway (clearance−centric, sleep–circadian, vascular–metabolic–immune) and diagnosis. To enhance transparency, **Table 1** summarizes human studies across conditions and MRI methods, and **Table 2** compiles methods/targets to enhance glymphatic clearance. We qualitatively appraised recurrent biases (head motion; sedation/sleep state; circadian timing; medication exposure, including “minimal exposure” in early psychosis; vascular/metabolic comorbidity; scanner/pipeline heterogeneity; cortisol assay timing; microbiome pre−analytics/analytics).

**Table 2:** Summarizes pharmacologic, behavioral, and device−based options highlighting targets, readouts, and psychiatric use−cases with putative glymphatic effects.

Lever/intervention	Primary target(s) on glymphatic pathway	Evidence level	Population/sample	Glymphatic proxy/readout	Clinical/biological correlates	Notes/psychiatric applicability
CBT−I; sleep consolidation	Increased deep NREM; decreased arousal/noradrenergic tone; increased vasomotion	Clinical evidence and mechanistic studies	Insomnia, MDD, mixed	BOLD−CSF coupling; sleep macro−architecture	Reduction in depression severity; improved cognition	Foundational lever in mood/psychosis with insomnia (6, 26).
Light−dark scheduling/chronotherapy	Circadian alignment; AQP4 polarization	Clinical practice + reviews	Mood disorders	Indirect (sleep/circadian markers)	Improved sleep timing, daytime function	Align med timing with clearance windows (88, 89).
Aerobic exercise	Increased vascular pulsatility; anti−inflammatory; astroglial support	Preclinical + human associative	Older adults; MDD	Indirect; DTI−ALPS where available	Memory/cognition benefits; anti−inflammatory	AQP4−dependent benefits in models (54).
Lateral sleep posture; slow breathing	Reduction in venous outflow resistance; entrain CSF with respiration	Preclinical + human physio	Healthy; patient groups	CSF flow surrogates	Better coupling of cardiac/respiratory drivers	Low−risk adjuncts (14, 16, 24).
rTMS (older adults)	Network−level modulation; sleep and vascular coupling	Early clinical signal	Older adults	“Glymphatic proxies” + cognition	Cognitive improvement	Mechanistic bridge to psychiatry; needs target−engagement biomarkers (90).
40 Hz multisensory (“gamma”) stimulation	Gamma entrainment favors neurovascular/meningeal coupling; Admeloriate paravascular clearance	Preclinical (mouse), high−impact	Amyloid mouse models	CSF/perivascular clearance; amyloid reduction	Improved behavioral readouts	Translational potential to psychiatric cohorts with disrupted sleep/network dynamics; human feasibility needed (91).
Melatonin	Circadian/AQP4 polarity; antioxidative	Preclinical + early clinical	Sleep loss, ICH models	AQP4 polarity; cognitive & BBB outcomes	Sleep alignment; cognitive benefit	Candidate chronobiotic in mood/PTSD (92–94).
Omega−3 PUFAs	Vascular/astroglial integrity	Preclinical + small clinical	Depression/cognition	Indirect; vascular markers	Cognitive & mood benefits	Pro−glymphatic vascular support (95, 96).
Lithium	Choroid plexus clock; CSF production dynamics	Preclinical/physiology	Bipolar disorder	Indirect (CP clock)	Mood stabilization	May influence day–night CSF rhythms (97).
Dexmedetomidine	NE−sparing sedation; increased intrathecal drug delivery; protect AQP4 pathways	Preclinical + peri−op studies	Surgical/ICU; TRD pilots	Tracer delivery; conceptual proxies	Antidepressant−like signals in TRD	Time−sensitive, “glymphatic−friendly” sedation candidate (98–100).
Ketamine/esketamine	NMDA modulation; astroglial effects	Preclinical mixed + clinical efficacy	Depression	Conflicting preclinical glymph findings	Rapid antidepressant effects	Mixed glymphatic signals (impair *vs* improve via astrocyte pyroptosis); consider dose/timing/context (101, 102).
Benzodiazepines; late−evening alcohol; zolpidem	Decreased REM & SWS quality; disrupt NE slow vasomotion	Observational/mechanistic	Insomnia; SUD	Sleep architecture; NE oscillations	Worsened sleep, cognition	Use sparingly/strategically; potential anti−glymphatic effects (39, 71, 103).

## 3 Characteristics of neuroimaging studies in patients with psychiatric pathology and clinical insights

### 3.1 Study landscape across diagnoses

**Figure 2:**
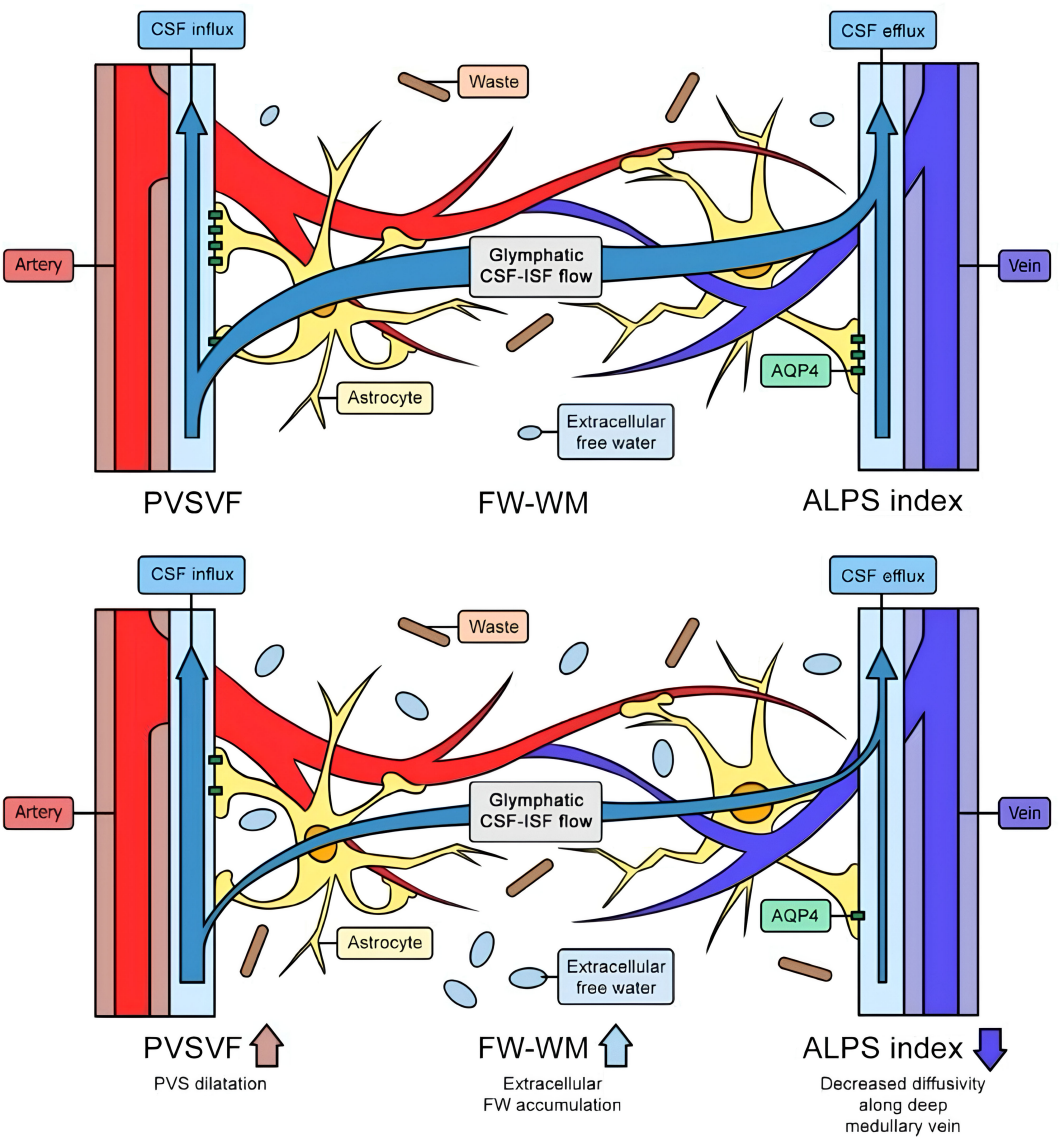
The top image illustrates a healthy glymphatic system, efficiently clearing waste and adapting to changing conditions. The bottom image shows chronic glymphatic dysfunction, characterized by a reduced ALPS index, dilated perivascular spaces, waste accumulation, extracellular fluid buildup, and decreased outflow. Legend: FW: free water; PVSVF: perivascular spaces venous flow; WM: waste matter; CSF: cerebrospinal fluid; ALPS: along perivascular spaces; AQP4: aquaporin 4; ISF: interstitial fluid.

Human neuroimaging studies that use diffusion‐MRI proxies of perivascular transport predominantly DTI−ALPS, BOLD–CSF coupling, or ASL consistently suggest glymphatic alterations across major psychiatric disorders **Figure 2**. In MDD, ALPS reductions co−occur with inflammatory and oxidative signatures and choroid plexus (ChP) changes, pointing to a CSF–immune–clearance axis (17, 62). Recent work also links lower ALPS indices to dysregulated diurnal cortisol secretion in major depressive disorder, highlighting a potential HPA–glymphatic coupling (104). An additional multicenter analysis indicates that glymphatic function moderates the link between peripheral inflammation and psychomotor retardation (PMR) in MDD, i.e., lower ALPS amplifies inflammation−related PMR and motor−network alterations (105). Notably, one large study in drug−naïve somatic depression reports higher ALPS (*vs* controls) during wakefulness, correlating with thalamic volume, interpreted as heightened daytime activity and thalamic vulnerability rather than overall “better clearance” (65). Preliminary preprint data also suggest that reduced ALPS values are associated with higher rumination severity and specific functional-connectivity patterns in major depressive disorder (106). In schizophrenia and early psychosis, ALPS reductions relate to cognitive deficits and are present in minimally medicated cohorts (60, 61). Emerging work links glymphatic metrics with gut dysbiosis and cognition, positioning the microbiome–meningeal–CSF axis as a candidate mechanism (107).

In BD, a diffusion−MRI study reported free−water increases, suggesting neuroinflammatory/extracellular fluid changes, without robust ALPS differences (35). Independent work associates frontal−pole atrophy with glymphatic dysfunction in BD (66).

In PTSD, higher cortical mean diffusivity in regions vulnerable to neurodegeneration correlates with lower ALPS (34). SUD show ALPS abnormalities and clinically meaningful ALPS–CBF coupling that aids classification in methamphetamine dependence (63), and ALPS–cognition associations in alcohol use disorder (73).

Neurodevelopmental disorders, ALPS reductions are reported in ASD and in ADHD, both children and adults, with links to age and cognition (75–77).

Insomnia with depression shows concurrent default mode network dysfunction and glymphatic alterations (108). A preliminary acute psychosis pilot study during early hospitalization suggests ALPS abnormalities in young adults (70).

### 3.2 Imaging readouts and their correlates

Most studies used DTI−ALPS with atlas−constrained ROIs; several adjusted for age, sex, education, and motion; fewer accounted for time−of−day or sleep. Consistent correlates include depression severity (HAMD), fatigue, cognition, systemic inflammation/oxidative stress indices (NLR, PLR, SII, etc.), and ChP volume (62). In major depressive disorder, ALPS reductions have also been linked to tract-specific white-matter alterations, reinforcing the connection between glymphatic metrics and microstructural integrity (109). In schizophrenia/psychosis, ALPS tracks cognitive impairment (61) and appears reduced even with minimal antipsychotic exposure (60).

### 3.3 Clinical insights and mechanistic threads

Convergent threads suggest that sleep–circadian disruption, vascular/IPAD changes, astroglial/AQP4 polarity, and immune–ChP signaling jointly shape glymphatic measures in psychiatric populations. Large−scale MDD data indicate ChP enlargement and lower ALPS with stronger ties to inflammatory ratios in patients than controls, consistent with a peripheral–central inflammatory bridge modulating CSF production and clearance (62). Conversely, when assessed during wakefulness, elevated ALPS in somatic−symptom MDD may reflect state−dependent disinhibition of daytime glymphatic activity (65).

### 3.4 Methodological notes

Medication exposure, time−of−day, sleep the night before scanning, and vascular risk vary across studies. Importantly, some reports include medication−naïve or minimally exposed cohorts (60, 65), strengthening causal inferences to the disease process. Multi−modal combinations, ALPS and CBF, enhance classification and may help disentangle vascular from perivascular drivers (63).

## 4 A glymphatic-oriented paradigm in psychiatric pharmacotherapy

Growing awareness of the glymphatic system raises the question of whether enhancing paravascular flow might optimize pharmacotherapy. Traditionally, psychiatric medications have been formulated and dosed with a primary emphasis on neurotransmitter or receptor targets. However, many agents linger in the CNS and often cause sedation or metabolic disturbances. In contexts of impaired glymphatic clearance, driven by insomnia, chronic inflammation, or circadian misalignment, drugs may accumulate or distribute unevenly, exacerbating lethargy and cognitive side effects (1, 2, 6, 49). By contrast, sedative strategies that conserve or deepen slow−wave sleep could expand extracellular spaces and support more efficient clearance of metabolic waste and residual drugs (15, 110). Interventions that bolster astrocyte functionality (e.g., anti−inflammatory strategies that stabilize AQP4 polarization) and circadian alignment, timing medications when glymphatic flow peaks (89, 111), are convergent levers that may improve both efficacy and tolerability (17, 26, 30, 88, 112).

Non−pharmacological levers complement medication strategies. Cognitive behavioral therapy for insomnia, structured light–dark exposure, and aerobic exercise improve sleep consolidation and vascular pulsatility, both key drivers of CSF–ISF exchange (6, 17, 30). Postural habits, in particular favoring lateral recumbency during sleep, and slow, regular breathing can also support perivascular transport (14, 16, 24). Emerging neuromodulation adds a mechanistic foothold: in older adults, repetitive transcranial magnetic stimulation (rTMS) has been reported to modulate putative glymphatic proxies alongside cognitive outcomes, suggesting a network−level pathway to “glymphatic−friendly” brain states translatable to psychiatric populations (90).

Network−level neuromodulation and sensory entrainment. Beyond rTMS, multisensory 40 Hz “gamma” stimulation, delivered via synchronized visual and auditory stimuli, has been shown in mouse models to promote glymphatic clearance of amyloid, likely by coupling neuronal oscillations to neurovascular dynamics and meningeal/lymphatic outflow pathways (91). Gamma entrainment reduced amyloid burden and improved behavioral readouts in preclinical settings, with concomitant signatures consistent with enhanced perivascular transport. While clinical translation to psychiatric cohorts remain exploratory, this approach could be mechanistically appealing for disorders marked by sleep fragmentation, neuroinflammation, and impaired network dynamics, as it could jointly influence vasomotion, arousal state, and astroglial physiology, all key determinants of glymphatic throughput. Early feasibility work in humans is needed to establish target engagement (e.g., BOLD−CSF coupling, DTI−ALPS) and symptom relevance.

Pharmacologic levers, dosing time, and sleep architecture. Melatonin may realign circadian timing and AQP4 polarity, while omega−3 PUFAs protect cerebrovascular and astroglial integrity, both plausibly pro−glymphatic and symptom−relevant in mood disorders (64, 92, 93, 95, 96, 113). Lithium’s effects on the choroid plexus clock hint at leverage via CSF production and day–night dynamics (97). Among sedatives, dexmedetomidine may be “glymphatic−friendly,” enhancing intrathecal drug delivery and potentially countering anesthetic−induced glymphatic disruption (98–100). By contrast, chronic benzodiazepine use, and late−evening alcohol may degrade sleep architecture and fluid transport, and zolpidem reduces norepinephrine slow−wave dynamics implicated in vasomotion and CSF movement, arguing for sparing, time−sensitive use (39, 71, 103). Ketamine shows mixed glymphatic effects across preclinical paradigms, both impairment and improvement have been reported, underscoring the importance of dose, timing, and disease context (101, 102). A preliminary, non– peer−reviewed report suggests that pairing esketamine with dexmedetomidine−based sleep modulation may accelerate antidepressant response and improve sleep in patients with depression and insomnia, a concept consistent with pro−glymphatic interventions but awaiting confirmation in randomized, peer−reviewed trials (114).

Collectively, “glymphatic−friendly” practice may include: prioritizing NREM−deepening over nonspecific sedation; aligning dosing to circadian windows of heightened clearance; supporting astrocyte health and vascular pulsatility; and integrating neuromodulatory or behavioral tools that stabilize network and autonomic dynamics central to perivascular flow. **Table 2** summarizes pharmacologic, behavioral, and device−based options highlighting targets, readouts, and psychiatric use−cases.

## 5 Conclusion

Viewing the brain as an organ that depends on nightly fluid clearance reframes psychiatric pathophysiology and care. Convergent evidence, from DTI−ALPS, CSF−BOLD and ASL coupling, choroid−plexus and inflammatory correlates, and preliminary interventional work, indicates that disrupted glymphatic function can perpetuate neuroinflammation, cognitive−affective symptoms, and suboptimal pharmacotherapy response in mood, psychotic, trauma−related, substance−use, and neurodevelopmental disorders (32, 34, 60, 62, 115). A glymphatic−oriented paradigm, preserving slow−wave sleep, aligning dosing to circadian biology, supporting astroglial and vascular health, and selectively leveraging agents such as dexmedetomidine, melatonin, and PUFAs while clarifying the role of ketamine and ensuring the judicious use of hypnotics, offers actionable paths to improve outcomes. Prospective trials that combine symptom scales with objective glymphatic readouts (e.g., ALPS, CSF−BOLD) are warranted to define who benefits, by how much, and with which combinations of behavioral, device−based, and pharmacologic interventions (17, 30, 90).
